# The Role of Cellular Glutathione Redox Cycle and Glutathione in the Regulation of Ileum Contractility

**DOI:** 10.3390/ijms27156920

**Published:** 2026-08-01

**Authors:** Tanja Grahovac, Zorana Oreščanin Dušić, Aleksandra Nikolić-Kokić, Duško Blagojević, Teodora Vidonja Uzelac

**Affiliations:** Department of Physiology, Institute for Biological Research “Siniša Stanković”—National Institute of the Republic of Serbia, University of Belgrade, 11000 Belgrade, Serbia; tanja.grahovac@ibiss.bg.ac.rs (T.G.); zoranaor@ibiss.bg.ac.rs (Z.O.D.); san@ibiss.bg.ac.rs (A.N.-K.)

**Keywords:** redox homeostasis, antioxidant enzymes, reduced glutathione, oxidized glutathione, carmustine (BCNU), rat, thiol, isolated organ bath

## Abstract

Redox homeostasis is driven by the ratio of the concentrations of cellular redox couples. The aim of this study was to reduce 2GSH/GSSG turnover in an ex vivo ileum by the irreversible inhibition of glutathione reductase (GR) activity (by BCNU) and evaluate its effects on contractility, antioxidant enzyme activity, and thiol levels. Increasing concentrations of BCNU as well as a single EC50 BCNU dose significantly reduced both contraction amplitude and GR activity. The addition of cumulative doses of both GSH and GSSG after EC50 BCNU caused further dose-dependent amplitude reduction, but turned GR activity to control levels and reduced CuZn-superoxide dismutase activity; cumulative doses of GSH also decreased catalase activity. Both GSH and GSSG cumulative doses decreased the contractility of non-treated control ileum in a dose-dependent manner, while GSH also increased glutathione peroxidase (GPx) activity. The correlation analysis showed a negative relationship between contractility and non-protein thiols, and positive correlations between catalase and GPx activities in BCNU treated ileum, as well as between GPx and non-protein thiols in solvent controls. The results present a framework for how glutathione turnover and ileum contractility are linked.

## 1. Introduction

In cells, the physiological balance between oxidative and reductive cellular capacity and processes is called redox homeostasis. Redox homeostasis is driven primarily by the ratio of the concentrations of important cellular redox couples such as NAD+/NADH (nicotinamide adenine dinucleotide), NADP^+^/NADPH (nicotinamide adenine dinucleotide phosphate), TrxSS/Trx(SH)2 (thioredoxin), protein sulfhydryls and finally 2GSH/GSSG (reduced/oxidized glutathione) as the most abundant redox couple in a cell [1]. This also represents the redox code as a set of principles that defines their positioning in space and time in biological systems [2]. The shift in NADH/NAD^+^ ratio and mediators that influence it define energetic cellular status and regulate smooth muscle contractility [3]. On the other hand, the 2GSH/GSSG couple is responsible for the biological status of the cell and is one of the main determinants of cellular redox state. It is known that significant cellular processes are driven by the GSH/GSSG ratio, such as oxygen sensing, and include different regulatory pathways such as hypoxia-inducible factors (HIFs), nuclear factor (NF)-kB, activator protein-1, heat shock factors, and p53, among others [4,5]. They depend on glutathione GSH/GSSG equilibrium [5,6,7,8]. In cells, a homeostatic 2GSH/GSSG equilibrium and ratio are obtained by the activity of glutathione reductase (GR), which reduces oxidized GSSG to GSH using NADPH (glutathione cellular redox cycle). However, the role of the glutathione redox cycle in the regulation of dynamic smooth muscle contractility is unknown, as is what happens when glutathione based redox homeostasis is disturbed by slowing down their turnover. Therefore, the first aim was to experimentally provoke slowed glutathione turnover in an ileum ex vivo by the inhibition of GR activity with BCNU (1-3-bis(2-chloroethyl)-1-nitrosourea; carmustine), and find out how this influences ileum smooth muscle contractility, antioxidant enzymes activity and total thiol concentration. BCNU is known as a dose- and time-dependent irreversible GR inhibitor [9,10,11,12] that easily penetrates into cells and exhibits its inhibitory effect comparatively fast. Irreversible GR inhibition in vitro occurs after the biochemical reduction of the enzyme in the presence of NADPH [12,13]. The second aim was to determine whether the addition of extracellular glutathione (either reduced or oxidized) could improve contractile activity, redox impairment provoked by BCNU on the level of antioxidant defense enzymes, and total cellular thiol concentrations. Oral glutathione supplements are available now, and marketed based on their effects on skin pigmentation [14] and the gut microbiome of type 2 diabetic individuals [15]; it is also slowly becoming nutraceutical. In line with this, our work seeks to explore further the role of principal thiols in smooth muscle contractility as well as in adequately maintaining redox balance in proper smooth muscle physiological functions. We previously showed the inhibitory role of hydrogen sulfide (H_2_S) and sodium sulfide as cellular synthetized physiologically active sulfides in smooth muscle contractility [16], as well as methanethiol [17], showing its different inhibitory kinetics and activity dose, which depended not only on thiol group, but also on the molecular structure and biological properties. Furthermore, their addition to isolated active uteri ex vivo induced a reduction in contractility, an imbalance in cellular redox state, and changes in the level of antioxidant enzymes’ activity [16,17], providing additional evidence of the coexistence and interdependence of redox state, antioxidant enzymes’ defense activity-based redox homeostasis, and smooth muscle contractility. Although both are thiol-based agents, they differ in their mechanisms of action, as well as in adaptive changes on the level of antioxidant enzyme activity, providing both stability in the redox state and smooth muscle contractility. However, the real nature of this interplay is still to be found, and far from fully understood. Now, it is glutathione’s turn.

Along with the measurement of contractility, the effects of the external addition of glutathione on the levels of antioxidant defense enzymes (SOD1—Copper-zinc superoxide dismutase, SOD2—Manganese superoxide dismutase, CAT—Catalase, GPx—Glutathione peroxidase and GR) were measured. It is known that cellular redox homeostasis is also achieved by the balanced production/elimination of reactive oxygen species (ROS) by essential antioxidant defense enzymes’ functional activity and overlapping connectivity. GR is considered as a part of the antioxidant defense, along with other antioxidant enzymes such as SOD, CAT and GPx, and here, they are used as (in)direct measures of the presence of cellular (pro)oxidative pressure and cellular inherent reactivity to (pro)oxidative challenge. Besides this, in this respect, the concentrations of different cellular thiols (–SH-containing molecules) also represent a part of the overall redox state [18]. They also undergo different oxidative modifications under oxidative stress [8] that could decrease both the amount of cellular GSH and protein sulfhydryl groups [19], and in turn affect overall cellular thiol concentration. The individual concentrations of specific protein thiols are far lower than those of the glutathione couple, and glutathione represents the major redox buffer in cells [20], although the concentration of total protein sulfhydryl groups is in general greater than that of GSH, and can aid in maintaining the service of the GSH pool. Therefore, in this work, we also measured both total and protein-containing cellular thiol content, as well as its protein to non-protein ratio, in order to determine how cellular glutathione turnover imbalance could be reflected in the amount of total free/bound thiols related to contractile activity.

## 2. Results

### 2.1. Effect of BCNU on Ileum Contractility and Antioxidant Enzyme Activities

**Figure 1:**
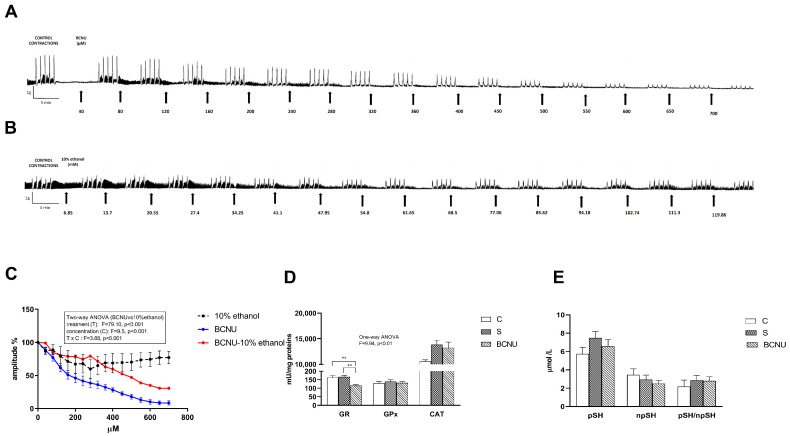
Ileum treated with increased concentrations of carmustine (BCNU). (**A**)—Representative original traces of electrically stimulated ileum treated with an increased concentration of BCNU. (**B**)—Representative original traces of electrically stimulated ileum treated with an increased concentration of 10% ethanol. (**C**)—Amplitude of ileum contraction (BCNU—blue solid line; BCNI-10% ethanol—red solid line and solvent (10% ethanol)—black dashed line). (**D**)—The activity of antioxidant enzymes, BCNU, solvent (S-10% ethanol) and control ileum (C) (GR—glutathione reductase, GPx—glutathione peroxidase, CAT—catalase). (**E**) Concentrations of –SH groups (pSH-protein –SH groups, npSH-non protein –SH groups). Data are expressed as the mean ± SEM. Statistical significance was tested by two-way (**B**) or one-way (**C**) ANOVA and post hoc compared by Tukey’s HSD test. ** *p* < 0.01.

The addition of increased concentrations of BCNU (Figure 1A,C) to an isolated and electrically stimulated ileum led to a statistically significant decrease in amplitude compared to controls (the addition of just the BCNU solvent (10% ethanol) in equal amounts that had been combined with dissolved BCNU used as a control) and after subtracting solvent-induced inhibition (two-way ANOVA treatment and concentration effects F = 79.1, *p* < 0.001, and F = 9.5, *p* < 0.001, respectively). The addition of the BCNU solvent with 10% ethanol reduced the amplitude (Figure 1B), but to an extent that was neither significant nor dose-dependent compared to BCNU (two-way ANOVA interaction effect F = 3.88, *p* < 0.001; post hoc Tukey’ unequal N HSD *t*-test, *p* < 0.001). However, to additionally clarify the effect of the solvent, a one-way ANOVA statistical analysis on the effect of adding only 10% ethanol on ileum contractility was performed, and the results show that there was no statistically significant effect of concentration (one-way ANOVA, solvent concentration effect, F = 0.575, *p* = 0.88). However, dissolving BCNU in 10% ethanol had an effect because the BCNU’s effect of inhibiting contractility after subtracting solvent-induced inhibition was significantly less pronounced compared to BCNU treatment without subtraction (ANOVA treatment effect, F = 79.1, *p* < 0.001, post hoc Tukey’s unequal N HSD *t*-test, *p* < 0.001). Treating with BCNU at concentrations above 550 µM significantly lowered the amplitude compared to non-treated and lower concentration doses, as well as controls (Tukey’s post hoc test, *p* < 0.001). The significance of the BCNU’s effects was confirmed by regression analysis that showed that BCNU’s effect of inhibiting contractility was dose-related (regression coefficient R^2^ = −0.688, F = 278, *p* < 0.001; intercept—75.5 ± 2.7, t = 28, *p* < 0.001; b = −0.111 ± 0.007, t = −16.7, *p* < 0.001). The same was obtained when regression analysis was applied on the effects of BCNU after subtracting solvent-induced inhibition (regression coefficient R^2^ = −0.468, F = 73, *p* < 0.001; intercept—98.8 ± 4.7, t = 83, *p* < 0.001; b = −0.68 ± 0.08, t = −8.5, *p* < 0.001).

The addition of BCNU significantly reduced GR activity (ANOVA F = 9.94, *p* < 0.01) compared to both non-treated and solvent-treated samples (Figure 1D). Despite lower GR activity, the amounts of both protein-bound and non-protein-bound –SH groups as well as their ratios were similar to both types of control (non-treated and solvent treated) ileum samples (Figure 1E) in the BCNU-treated ileum.

Adding 10% ethanol as the solvent for BCNU increased the CAT activity compared to non-treated controls, but was not significantly different from BCNU, meaning that BCNU had no effect on the CAT level (Figure 1D).

Correlation analysis (Table 1) showed that, in BCNU-treated samples, amplitude contractility negatively correlated with the amount of non-protein –SH groups (*p* < 0.05). There is also a positive correlation between the levels of CAT and GPx activities in the same group (*p* < 0.05). In the group treated only with BCNU solvent (10% ethanol), there is a positive correlation with GPx activity and the levels of non-protein –SH groups (*p* < 0.05).

### 2.2. Effects of Reduced and Oxidized Glutathione on Contractility and Antioxidant Enzyme Activities in BCNU Pre-Treated Ileum

**Figure 2:**
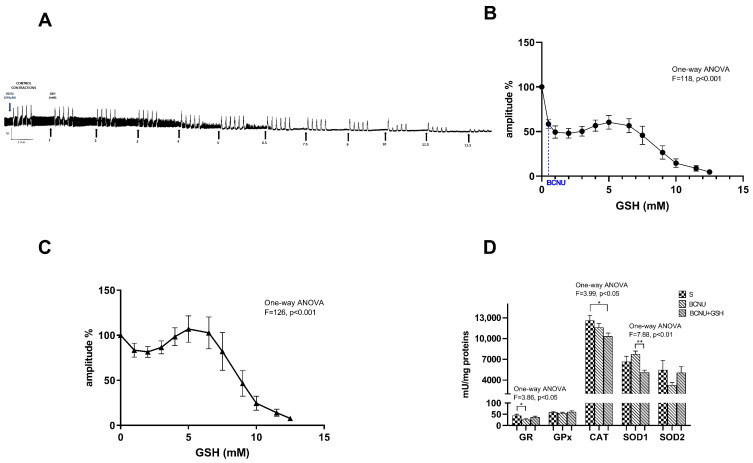
Ileum pretreated with the EC50 (half maximal effective concentration) of BCNU and further treated with increased cumulative doses of GSH (reduced glutathione). (**A**)—Representative original traces of electrically stimulated ileum pretreated with the EC50 of BCNU and further treated with increased cumulative doses of GSH. (**B**)—Amplitude of ileum contraction pretreated with the EC50 of BCNU and further treated with increased cumulative doses of GSH (control non-treated ileum contraction taken as 100%). (**C**)—Amplitude of ileum contraction pretreated with the EC50 of BCNU and further treated with increased cumulative doses of GSH (EC50 of BCNU effect taken as 100%). (**D**)—The activity of antioxidant enzymes: S-ileum treated with solvent (10% ethanol), BCNU ileum treated with EC50 BCNU and BCNU + GSH-ileum pretreated with BCNU and further treated with increased cumulative doses of GSH, GR (glutathione reductase), GPx (glutathione peroxidase), CAT (catalase); SOD1 (copper–zinc superoxide dismutase), SOD2 (manganese superoxide dismutase). Data are expressed as the mean ± SEM. Statistical significance was tested by one-way ANOVA and post hoc compared by Tukey’s HSD test. * *p* < 0.05; ** *p* < 0.01.

**Figure 3:**
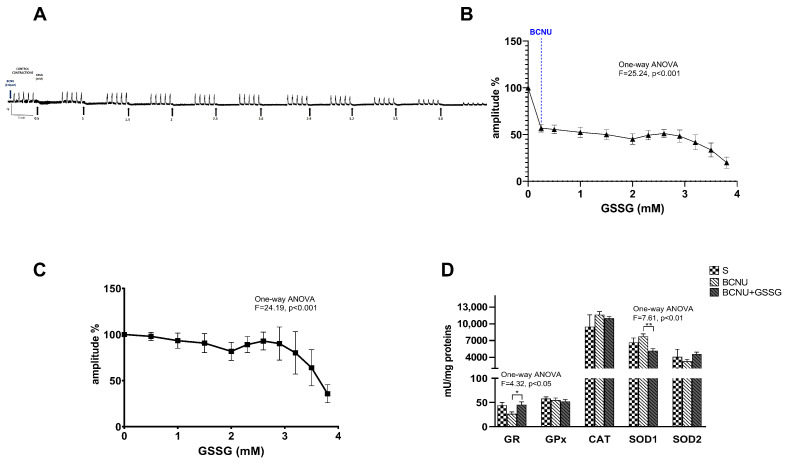
Ileum pretreated with the EC50 (half maximal effective concentration) of BCNU and further treated with increased cumulative doses of GSSG (oxidized glutathione). (**A**)—Representative original traces of electrically stimulated ileum pretreated with the EC50 of BCNU and further treated with increased cumulative doses of GSSG. (**B**)—Amplitude of ileum contraction pretreated with the EC50 of BCNU and further treated with increased cumulative doses of GSSG (control non-treated ileum contraction taken as 100%). (**C**)—Amplitude of ileum contraction pretreated with the EC50 of BCNU and further treated with increased cumulative doses of GSSG (EC50 of BCNU effect taken as 100%). (**D**)—The activity of antioxidant enzymes: S—ileum treated with solvent (10% ethanol), BCNU—ileum treated with EC50 BCNU and BCNU + GSSG—ileum pretreated with BCNU and further treated with increased cumulative doses of GSH, GR (glutathione reductase), GPx (glutathione peroxidase), CAT (catalase), SOD1 (copper–zinc superoxide dismutase), and SOD2 (manganese superoxide dismutase). Data are expressed as the mean ± SEM. Statistical significance was tested by one-way ANOVA and post hoc compared by Tukey’s HSD test. * *p* < 0.05; ** *p* < 0.01.

The pre-treatment with one EC50 (half maximal effective concentration) BCNU dose led to an initial decrease in the ileum’s amplitude after electrical stimulation to 57 ± 12 percent of the control amplitude after the stimulation (Figure 2B and Figure 3B). Further treatment with increased cumulative doses of GSH led to a dose-dependent (Univariate Tests of Significance, F = 118, *p* < 0.001; partial correlation coefficients R = −0.747 ± 0.07, *p* < 0.001) decrease in amplitude that was statistically significant for doses 9 mM and more (one-way ANOVA dose effect F = 20.85, *p* < 0.001; Tukey’s post hoc HSD test, *p* < 0.001) (Figure 2A,B). The same is true for GSSG; cumulative doses of GSSG led to further dose-dependent (Univariate Tests of Significance, F = 25.24, *p* < 0.001; R = −0.476 ± 0.09) decreases in amplitude that became significantly different from BCNU EC50-treated amplitude only with concentrations of 3.8 mM (one-way ANOVA dose effect F = 4.81, *p* < 0.001, Tukey’s post hoc HSD *t*-test, *p* < 0.001) (Figure 3A,B).

However, when the effects of the addition of glutathione at cumulative concentrations were normalized to the EC50 BCNU effect as 100%, the results (Figure 2C) show that GSH led to a dose-dependent (Univariate Tests of Significance, F = 126, *p* < 0.001; R = −0.76 ± 0.07) decrease in amplitude that was statistically significant for doses 9 mM and more (ANOVA F = 23.38, *p* < 0.001; Tukey’s post hoc HSD test *p* < 0.001). The same held for GSSG—treatment with increased cumulative doses of GSSG led to dose-dependent amplitude reductions (Univariate Tests of Significance, F = 24.19, *p* < 0.001, R = −0.47 ± 0.1) with concentrations of 3.8 mM that led to significant decreases in amplitude compared to BCNU EC50 controls (ANOVA dose effect, F = 4.567, *p* < 0.001; Tukey’s post hoc HSD test, *p* < 0.001) (Figure 3C).

The activities of antioxidant enzymes in ileum samples treated with single EC50 BCNU doses upon cumulative additions of either GSH or GSSG were compared to those of control samples (which were given single doses of BCNU solvent 10% ethanol). The addition of BCNU decreased the GR activity in ileum samples (one-way ANOVA F = 3.86, *p* < 0.05) compared to controls (post hoc Tukey’s Unequal N HSD *t*-test, *p* < 0.05), but GSH addition restricted the GR activity to that of the controls (Figure 2D). The CAT activity in ileum samples after the addition of single doses of EC50 BCNU was not statistically significant compared to controls, but the addition of cumulative GSH doses decreased CAT activity (one-way ANOVA F = 3.99, *p* < 0.05; Tukey Unequal N HSD post hoc *t*-test, *p* < 0.05) to below the control level. BCNU EC50 dosage slightly increased SOD1 activity, but to a level that was not statistically significant in comparison with controls, and the further addition of GSH decreased SOD1 to a level below controls; statistical significance was again only recorded for BCNU EC50-treated samples (one-way ANOVA, F = 7.88, *p* < 0.01; Tukey Unequal N HSD post hoc *t*-test, *p* < 0.01). The treatment of ileum samples with single EC50 BCNU doses had no effects on either SOD2 or GPx activities (Figure 2D).

There were no differences among the levels of the CAT, SOD2 and GPx antioxidant enzymes’ activities after either pre-treatment with BCNU EC50 or after the addition of cumulative doses of oxidized glutathione compared to corresponding controls (Figure 3D). However, the addition of a single BCNU EC50 dose reduced the GR level compared to corresponding controls (ANOVA, F = 4.32, *p* < 0.05; Tukey’s post hoc *t*-test, *p* < 0.05), but the addition of cumulative GSSG doses reduced the GR activity level to that of the control. The BCNU EC50 dosage slightly increased SOD1 activity, but to a level that was not statistically significant in comparison with controls. However, the further addition of GSSG decreased the SOD1 level to below that of controls, but statistical significance was recorded only for BCNU EC50-treated samples (one-way ANOVA, F = 7.61, *p* < 0.01; Tukey Unequal N HSD post hoc *t*-test, *p* < 0.01 (Figure 3D)).

### 2.3. Effect of Reduced and Oxidized Glutathione on Ileum Contractility and Antioxidant Enzyme Activities

**Figure 4:**
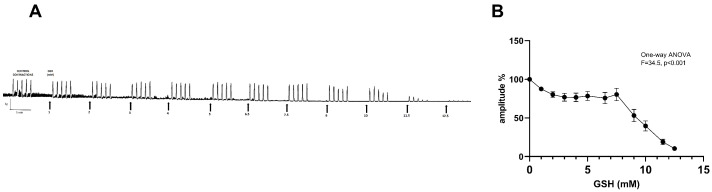
Ileum treated with increased cumulative doses of reduced glutathione (GSH). (**A**)—Representative original traces of electrically stimulated ileum. (**B**)—Amplitude of ileum contraction. Data are expressed as the mean ± SEM. Statistical significance was tested by one-way ANOVA and post hoc compared by Tukey’s HSD test.

Reduced glutathione decreased the amplitude of electrically stimulated isolated ileum samples (ANOVA F = 34.5, *p* < 0.001) in a dose-dependent manner (model significance F = 176, *p* < 0.001, regression coefficient, R = −0.72 ± 0.05, *p* < 0.001). The dose of 9 mM was enough to provoke a statistically significant reduction in amplitude that progressed with the increase in dose (post hoc Tukey’s *t*-test, *p* < 0.05, Figure 4A,B).

**Figure 5:**
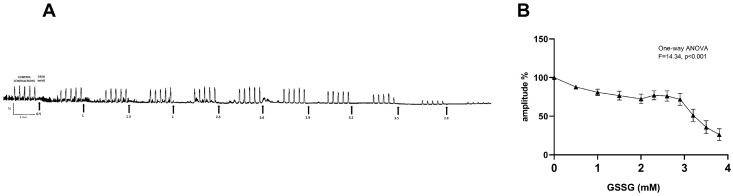
Ileum treated with increased cumulative doses of oxidized glutathione (GSSG). (**A**)—Representative original traces of electrically stimulated ileum. (**B**)—Amplitude of ileum contraction. Data are expressed as the mean ± SEM. Statistical significance was tested by one-way ANOVA and post hoc compared by Tukey’s HSD test.

Oxidized glutathione also decreased the amplitude of electrically stimulated isolated ileum samples (ANOVA F = 14.34, *p* < 0.001) in a dose-dependent manner (model significance F = 63.5, *p* < 0.001, regression coefficient, R = −0.54 ± 0.07, *p* < 0.001), with the dose of 3.5 mM the lowest that produced statistically significant amplitude reductions (post hoc Tukey’s *t*-test, *p* < 0.001, Figure 5A,B).

**Figure 6:**
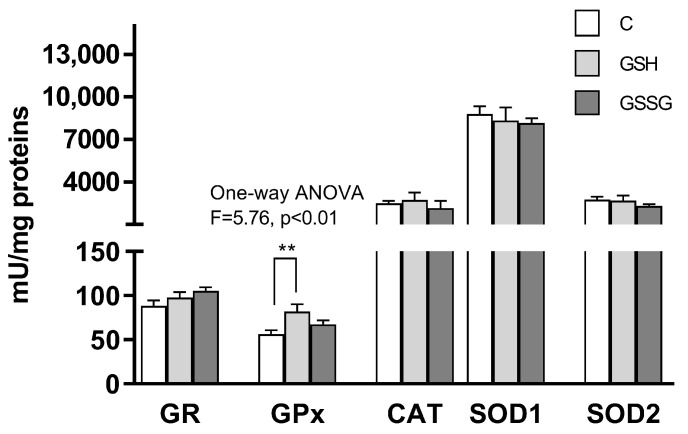
The activities of antioxidant enzymes in electrically stimulated ileum (control—C) and those treated with increased cumulative doses of reduced glutathione (GSH) or oxidized glutathione (GSSG). GR—glutathione reductase, GPx—glutathione peroxidase, CAT—catalase, SOD1—copper–zinc superoxide dismutase, SOD2—manganese superoxide dismutase. Data are expressed as the mean ± SEM. Statistical significance was tested by one-way ANOVA and post hoc compared by Tukey’s HSD test. ** *p* < 0.01.

Although the control ileum samples were used separately, the levels of antioxidant enzymes were not significantly different (no significant differences after ANOVA or paired *t*-test); therefore, controls were merged into one common control group. The addition of GSH to isolated ileum elevated the GPx (ANOVA F = 5.76 (*p* < 0.01) Tukey’s post hoc, *p* < 0.05) activity compared to non-treated controls (Figure 6). The addition of GSSG had no effects on the levels of antioxidant enzymes in GSSG-treated ileum samples.

Correlation analyses show that amplitude levels at the end of glutathione treatment do not statistically significantly correlate with the levels antioxidant enzymes in GSH- or GSSG-treated ileum samples (Table 2).

## 3. Discussion

In this paper, we challenged cellular redox homeostasis by the partial inhibition of GR activity, which led to slower oxidization and to reduced glutathione turnover, and we saw this reflected in specific physiological functions, this time specifically ileum (smooth muscle) contractility and antioxidant enzymes’ activity, as well as the concentration of cellular thiols. We observed that the physiological response to the disturbed turnover of the most concentrated redox couples that play a role in determining the cellular redox environment (2GSH/GSSG) in the case of an electrically stimulated ileum was a reduction in contractility without compensatory changes in the activities of the antioxidant enzymes (SOD, CAT, GPx) and the concentrations of thiols (both free and protein-bound). However, our interpretation of the effects of GSH/GSSG on this level is not complete, as both GSH and GSSG reduce the contractility even in an untreated ileum, suggesting that the inhibitory effect is not specific to glutathione redox cycling or GR inhibition.

The starting point of our experiment was to reduce GR activity and slow down glutathione turnover by BCNU. After treatment with cumulative concentrations of BCNU, the GR activity at the end of treatment was lowered to about 70% that of the controls. However, BCNU treatment also led to the inhibition of electrically stimulated contractility in the ileum. The BCNU solvent (10% ethanol) itself had an effect on contractility, but only in the beginning of the treatment (reduction of up to the 60% maximum at the corresponding concentration of 280 μM BCNU), with some recovery later (up to 80% at the corresponding concentration of 400 μM BCNU) that remained stable until the end of experiment. It is known that ethanol in small concentrations reduces spontaneous and stimulated ileum contractions via mechanisms similar to muscarinic blocker atropine [21]. Anyway, the ethanol concentrations applied in our experiment as the BCNU solvent were far lower and statistically insignificant. On the other hand, BCNU treatment led to concentration-dependent ileum contractility inhibition without significant fluctuations. The same effect was seen when BCNU’s effects were calculated after subtracting solvent-induced inhibition, but less pronounced. Taken together, it can be concluded that BCNU inhibited electrically stimulated ileum contractions, along with the minor influence of ethanol. Treatment with BCNU had no effects on the activities of other antioxidant enzymes, except GR. The effect of the solvent on the level of antioxidant activity was also measured, and it was found that the CAT activity slightly increased (*p* < 0.05) compared to non-treated controls, but the comparison of a BCNU-treated ileum with that treated with BCNU solvent controls (10% ethanol) revealed no change. Therefore, using a small amount of alcohol as a solvent had an influence on the experiment, but not to such an extent as to compromise either our model system based on BCNU or our conclusions. Furthermore, the measurement of antioxidant activity showed that BCNU treatment led to significant decreases in GR activity—up to 30% compared to both non-treated controls and those treated solely with BCNU solvent (10% ethanol). A reduction by 30% represents the significant slowdown of glutathione turnover that was our first experimental goal. The same was seen when one single EC50 BCNU dose was applied. One EC50 dose induced a drop in the amplitude of the electrically stimulated ileum (to 60% of control amplitude) and GR activity (to almost half of the control activity) without compensatory changes in either the activities of other antioxidant enzymes or the concentrations of total protein-bound and free thiols, as well as their ratio. At this point, we can conclude that slowing down the glutathione turnover by up to 30% is tolerable for the ileum smooth muscle as regards antioxidant enzyme protection, since there were no compensatory changes. However, we did see a reduction in contractile activity that could have been a consequence of BCNU treatment itself, and/or slowed glutathione turnover. There are not many data regarding BCNU’s direct pharmacological activity. BCNU is classified as an anticancer drug that acts as a DNA-alkylating agent, causing crosslinks in DNA strands and RNA, and thus preventing DNA replication and transcription, and the inhibition of DNA synthesis, RNA production and RNA translation (protein synthesis), inducing cell death without explicit other pharmacological activities [22]. There are reports that the BCNU treatment of PC12 cells can induce an influx of Ca^2+^ from extracellular Ca^2+^ via L-type voltage-gated calcium channels, which can be prevented by the blockade of Cl^−^ channels [23,24]. The presence of extracellular Ca^2+^, however, does not seem to be required to allow the BCNU-induced changes in Ca^2+^ permeability of the cell membrane, but it does enhance this effect, since there was no evidence of Ca^2+^ release from intracellular stores when carmustine was applied to PC12 cells bathed in the Ca^2+^-free extracellular solution. However, the increase in Ca^2+^ was delayed, and could be blocked, but only if L-type voltage-gated calcium channels are applied simultaneously with BCNU. The BCNU-mediated Ca^2+^ increase was enhanced when glutathione synthesis was blocked, but it was suppressed by iron chelating. This indicates that the mechanism of action of BCNU is redox, which regulates different types of receptors and requires iron. The iron further induces ROS production that cannot be suppressed due to low glutathione turnover and accumulated GSSG. The brief exposure of endothelial cells to BCNU greatly potentiated the activation of nonselective cation channels by organic peroxide [25], and increased the basal level of cytoplasmic Ca^2+^ [26]. However, cytoprotection by different multivalent cations had limited effectiveness in blocking hydrogen peroxide-activated nonselective cation channels in endothelial cells [27], contrary to the effects of the iron chelator deferoxamine [24], suggesting that ROS, especially hydrogen peroxide and/or organic peroxide, mediated BCNU’s effects. The incubation of brain slices with 1 mM BCNU for 2 h decreased GSH levels by >70%, and under these conditions the carbonylation of proteins increased by 2–3 fold. Again, the iron chelator deferoxamine, the superoxide scavenger rutin and the hydrogen peroxide (H_2_O_2_) quencher dimethylthiourea all prevented DEM-induced protein carbonylation and lipid peroxidation, indicating that the underlying mechanism involves the iron-catalyzed generation of hydroxyl radicals from H_2_O_2_ (Fenton reaction). Our previous results show that hydrogen peroxide induced the dose dependent inhibition of uterine contractions via the involvement of different types of receptors; among these, Kv channels played the most significant role [28]. Hydrogen peroxide targets a cysteine residue in the modulatory subunit of Kv channels that confers redox-sensitivity to the channel [29,30]; in a vascular thiol, this targets activating Kv-channels, leading to subsequent relaxation [31]. Regarding lipid peroxides, Park et al. [32] showed that BCNU-induced elevated GSSG can cause neural HT4 cell death in a 12-Lipoxigenase-sensitive manner. In fact, GSSG presence significantly increases the catalytic function of 12-lipoxigenase. Under such GSH-depleted conditions, HT4 cells were noted to contain significant amounts of S-glutathionylated proteins (and become more sensitive to GSSG-induced cell death), and that small amounts of arachidonic acid were lethal. Altogether, there are several potential mechanisms by which BCNU can interfere with signaling pathways, including ROS, both forms of glutathione and thiols. However, there were no effects on the levels of other antioxidant enzymes suggesting GR as a target, but we did see slower glutathione turnover and possibly different thiols that cannot be viewed as changes in total concentration. This also suggests that not just concentration, but also GSH/GSSG turnover and ratio, are important for cellular homeostasis in excitable tissues. Interestingly, the reduction in lipid peroxidation in rat liver microsomes revealed the synergistic effects of GSH and GSSG, which were concentration dependent [33]. Moreover, the cytosolic injection of GSSG was observed to selectively compromise mitochondrial membrane potential, but not to affect plasma membrane potential. This also suggests mitochondria as a target that is sensitive to changes in the GSH/GSSG turnover and ratio induced by BCNU. In rat heart mitochondria, BCNU shifted the cellular milieu toward more oxidative and increased mitochondrial H_2_O_2_ production without altering respiration, also causing a decrease in H_2_O_2_ removal by 57%. Furthermore, BCNU was not able to reduce the thioredoxin reductase activity or total (GSH + GSSG) glutathione level, but enhanced the level of GSSG [34]. The consequence of a lower available GSH level is that hydrogen peroxide removal by GPx is compromised. However, in our experiment, there were no changes in either CAT or GPx activity, suggesting that overall redox changes and disturbances were not of the magnitude or in the time frame that would cause significant compensatory changes in the enzyme activity or the amounts of cellular thiols, but instead occurred on the subtle level of several different signal points that depend on either GSH or GSSG. The correlation analysis of the enzyme activities of control and BCNU-treated electrically stimulated ilea and their contractile activity levels (expressed as peak height) confirmed this. Specifically, the amplitude levels were significantly negatively correlated with non-protein-bound (free) thiols (r = −0.735, *p* < 0.05) in the BCNU-treated ileum, which means that different –SH groups impair amplitude levels and gain additional importance, especially under conditions of reduced glutathione turnover. This confirms our previous result that small thiols inhibited uterine contractions in a dose-dependent manner [16,17], but with different kinetics and mechanisms of action. On the other hand, in the relevant ileum control, the correlation analysis revealed a positive significant correlation between GPx and non-protein-bound –SH (mainly representing GSH), which is in accordance with steady-state antioxidant and redox regulation. Regarding antioxidant activity, glutaredoxin reduction can also be impaired by BCNU-induced GR inactivity. BCNU is, on the other hand, ineffective on thioredoxin reductase [34]. It is possible that ileum antioxidant protection is affected by other cellular antioxidants, and both BCNU and glutathione’s effects manifest on discrete signaling levels.

After we had determined a reduction in glutathione turnover caused by BCNU, the next step was to try to establish a balance by adding glutathione externally. A single BCNU dose (EC50 extracted from the previous treatment) was applied, and this was followed by the cumulative addition of either GSH or GSSG. As expected, a single BCNU dose induced an inhibition of ileum contractility to 57% of the control amplitude after electrical stimulation. However, the addition of both GSH and GSSG led to further inhibitions of electrically stimulated contractility. The contractions that had conclusive effects could be compared as ≈2:1. Bearing in mind that GSSG had two polypeptide GS moieties, it seems that the effects led to similar numbers of GS moieties. At the level of the antioxidant enzymes, the addition of either GSH or GSSG caused the GR activity to match that of the non-treated controls, with a slight reduction in SOD1 activity that suggested the cytoplasm was the source of ROS generation and that the superoxide was the mediator of BCNU’s effects, since the addition of either GSH or GSSG to the non-treated control samples had no effect on SOD1 activity. The increase in GR activity could be a simple consequence of the elevation of the available glutathione concentration, which accelerates turnover due to the GR enzyme’s catalytic mechanism (the addition of glutathione accelerates the reaction kinetics of uninhibited molecules and elevates the reaction rate—this is the linear phase of enzyme kinetics), although some posttranslational modifications cannot be ruled out. The addition of GSH to the BCNU-treated ileum along with SOD1 reduction led to a reduced CAT activity, which could also have led to a slower release of hydrogen peroxide given the lower SOD1 activity. The differences in the reaction of CAT to GSH and GSSG could be consequences of the molecular location where external glutathione acts in BCNU-mediated molecular events. However, after the addition of GSH or GSSG to the BCNU-treated ileum, there was no improvement in contractility, but on the contrary, there was further inhibition. This suggests that the inhibitory effect is not specific to redox cycling or GR inhibition, but does have some direct glutathione role. Therefore, to compare and determine how glutathione itself is related to contractility, we exposed a control, non-treated ex vivo ileum to cumulative additions of either GSH or GSSG. The results show that both GSH and GSSG induced the dose-related inhibition of amplitude and contractility. Oxidized glutathione had higher inhibitory potential, since a much lower concentration was needed to reach complete ileum contractility inhibition (12.5 vs. 3.8 mM). Regardless, our results show that contractility inhibition was an effect of the exposure to external glutathione concentrations ranging around the mM level, regardless of the redox state. The inhibition of contractility seems to be characteristic of different forms of –SH-bearing thiols. The most molecularly basic thiol, H_2_S, is a well-known signal transmitter that expresses its activity via a DIDS-sensitive Cl pathway [16]. Components of the relaxation are also redox- and Ca^2+^-dependent. For small thiols, it seems that polysulfides, which act as oxidizing species, could be mediators that induce the S-sulfhydration of proteins [35]. On the other hand, the effects of GSH/GSSG are expressed by thiol–disulfide exchange. However, our results show no changes in the concentrations of cellular thiols, suggesting that all that changes are the targets, and these only take place at low levels that do not have an effect on the concentrations of total cellular thiols. Regardless, the pharmacological characterization of the receptors involved in glutathione-mediated electrically stimulated ileum contractility is the next target. It is unclear whether thiols (and glutathione) act as a direct muscle relaxant (on the smooth muscle) or as a modulator of neuronal activity (since the drug is an ileum with intact nerve plexuses). In an ex vivo electrically stimulated isolated ileum, signal transmission is undertaken by muscarinic acetylcholine receptors, where receptor signaling generates ROS, and the redox state of the receptor dictates its function by redox-sensitive cysteine modifications. The signal is further carried to G-protein coupled receptors (GPCRs), with its own active sites and structure that contain multiple cysteine residues that are highly sensitive to the cellular redox state. The oxidation of these thiol groups modifies the sterically binding site, changing the receptor’s affinity for acetylcholine and then regulating the contractile activity and the intensity of contractions. Moreover, the ROSs produced everywhere in the cell (such as hydrogen peroxide) can diffuse according to their chemical nature and reactivity, and express compartmentalized effects. Lipid peroxides from the membrane can also cause oxidative modifications of the M3 muscarinic receptor. Another possible influence is nitric oxide modulation through S-nitrosylation. However, all these require further experimental clarification, and constitute a phenomenon that opens up the way for further research. Our results show the importance of redox balance and glutathione turnover, but the reductions in contractility caused by both GSH and GSSG, even in an untreated ileum, suggest that the inhibitory effect is not specific to glutathione turnover or GR inhibition, but these do influence it.

Redox homeostasis is not stable; it is set by the redox set point, but permanently attacked by different free radicals and/or reactive species and is mainly driven by inherent internal production (mainly superoxide and both hydrogen and organic peroxides). The cellular counterbalance is organized and the cells are defended by cellular antioxidant defense mechanisms—a set of enzymes (superoxide dismutase, catalase, glutathione peroxidase and glutathione reductase being the main ones), low molecular antioxidants and thiols. This short-term homeostatic mechanisms of stress and stress response are considered as non-transcriptional, although there are also redox-responsive signaling molecules (NFkB, Nrf2/Keap1, HIF) that operate on constant alert by coupling oxidant and electrophile status to their level of activation [36]. The glutathione cellular redox cycle is also part of cellular antioxidant defense, since GSH is the reductive agent that provides electrons with the activity of the glutathione peroxidase enzyme family, which metabolizes hydrogen peroxide and lipid peroxides to H_2_O and corresponding alcohols. Increases in the production of reactive oxygen species thus lead to GSH’s oxidation to GSSG and thus shifts in the GSH/GSSG ratio that influence cellular redox homeostasis. Glutathione’s reductase activity maintains GSH/GSSG’s optimal cellular ratio by reducing GSSG with the assistance of NADPH as the reduced cofactor, enabling dynamic but stable cellular homeostasis despite metabolic ROS challenges. In our work, using BCNU, we challenged redox homeostasis on the level of impaired glutathione turnover and reduction, and showed how these important biological redox couples’ turnovers contribute to the regulation of ileum contractility. The inhibition of GR activity up to 50% (as well as glutathione turnover) is tolerable at the level of the main antioxidant enzymes’ activities (SOD, CAT, GPx) and the concentrations of free and protein-bound –SH groups, as well as their ratios. This suggests that in contractile tissue, the dominant redox factors are related to the redox signaling pathways. Moreover, the addition of external oxidized glutathione had no effects on antioxidant enzymes’ activity, but did have effects on contractility. On the other hand, the addition of GSH increased GPx activity, which could be due to the GPx enzyme’s kinetics that mean that increases in the substrate in the linear range led to higher activities. Regardless, some posttranslational modifications cannot be ruled out. However, our results offer a general framework of how the turnover and the maintenance of the GSSG/2GSH couple and ileum contractility are linked. However, the interpretation of GSH/GSSG’s effects is not complete, as both GSH and GSSG reduce contractility even in the untreated ileum, suggesting that the inhibitory effect is not specific to redox cycling or GR inhibition. Since some thiols might be considered as physiologically active factors and redox sensors that act through protein-bound thiol residues and their oxidation (especially of cysteine), which kinetically determine the pathways of molecular signaling and life cycles [8,37,38], the further characterization of the receptors involved in the glutathione regulation of contractility is necessary.

## 4. Materials and Methods

### 4.1. Experimental Animals

All procedures were conducted according to the guidelines of the EEC Directive on the Protection of Animals Used for Experimental and Other Scientific Purposes, and were approved by the Ethical Committee for the Use of Laboratory Animals of the Institute for the Biological Research “Siniša Stanković”—National Institute of the Republic of Serbia, University of Belgrade, Decision No. 000169330 2024 14841 002 001 000 001. Male *Wistar* rats were housed in the animal facility of the Institute for Biological Research “Siniša Stanković” under a standard dark–light cycle of 12 h/12 h and at room temperature, 22 °C. All rats had ad libitum access to food and tap water up to 16 h before the experiment, when they were only deprived of access to food. Four-month-old rats, with body mass 394 ± 8 g, were killed by rapid decapitation.

### 4.2. Isolated Organ Bath Studies

After decapitation, the rat ileums were rapidly excised and cleaned of surrounding connective tissue. The ileum was then cut into 4 strips approximately 1 cm long and mounted vertically in 10 mL organ baths (TSZ-04-E four-channel isolated organ bath system, MDE Research, Heidelberg, Germany) filled with Tyrode’s solution aerated with a mixture of 95% oxygen and 5% carbon dioxide at 37 °C. The Tyrode solution enables ileum contractility for hours with no fluctuations in pH and osmolality. Each strip was subjected to a constant 1 g weight tension. The contractile responses of the ileum strips were induced by electrical stimulation with frequency 16 Hz, pulse width 3 ms, pulse period 62.5 ms, train number 160, train delay 60 s and voltage 90 V. The ileum strips were given a 5-min pause between each package of electrical stimulation. After an equilibration period and before any treatment, the ileum strips were electrically stimulated, and the contractile activity measured here served as the non-treated control (100% contractile activity). In the first set of experiments, isolated ileum strips were treated with cumulative concentrations of BCNU dissolved in 10% ethanol (concentrations of 40, 80, 120, 160, 200, 240, 280, 320, 360, 400, 450, 500, 550, 600, 650 and 700 μM) along with internal controls and 10% ethanol in equal concentrations to those used during the BCNU treatment. The concentration of 10% of ethanol was the minimal concentration able to provide for the dissolution of BCNU in water in concentrations that could further allow us to perform the experiment. The second experiment was performed on two groups of ileum strips pre-treated with a single EC50 dose of carmustine that was determined in the first set of experiments, and then treated with increasing concentrations of either GSH or GSSG. The electrically stimulated ileum strips were left in isolated organ baths without any treatment and monitored for as long as other groups that were treated, and they served as controls. In the third set of experiments the effects of GSH and GSSG on ileum contractility were studied by individual cumulative addition up to complete contractility inhibition. One group of ileum strips were treated with increasing concentrations of GSH (concentrations of 1, 2, 3, 4, 5, 6.5, 7.5, 9, 10, 11.5 and 12.5 mM), and the ileum strips were electrically stimulated immediately after each dose. Another group of ileum strips was treated with increasing concentrations of GSSG (concentrations of 0.5, 1, 1.5, 2, 2.3, 2.6, 2.9, 3.2, 3.5 and 3.8 mM), and 5 min after each dose was applied, they were electrically stimulated. Since GSH and GSSG were dissolved in dH_2_O, the ileum strips were electrically stimulated in isolated organ baths without any treatment and monitored for as long as other groups, and these served as controls. After isolated organ bath treatment, the ileum strips were frozen in liquid nitrogen and transferred to −80 °C, until the measurement of the antioxidant enzymes’ activities.

### 4.3. Measurement of Antioxidant Enzyme Activities and Sulfhydryl Groups

In order to prepare the samples for further procedures, frozen ileum strips were thawed, chopped, homogenized (3 × 10 s) and sonicated (3 × 15 s) in 0.25 M sucrose, 0.05 M Tris and 1 mM EDTA cold buffer with pH 7.4. All procedures were performed on ice. Sonicates were than centrifuged for 90 min at 105.000× *g* at 4 °C (OptimaTM L-100 XP Ultracentrifuge, Beckman Coulter, Brea, CA, USA). The supernatants were used for determining total protein concentration and for the spectrophotometric measurement of antioxidant enzyme activities and sulfhydryl groups.

The total protein concentration was measured according to the Lowry method [39] modified for microtiter plates, based on the reaction of Cu+, produced by the oxidation of peptide bonds with Folin–Ciocalteu reagent. The reaction causes a color change in the solution with absorption in the range of 650 to 750 nm, detectable with a spectrophotometer (Multiskan Spectrum, Thermo Fisher Scientific Oy, Vantaa, Finland).

The CAT activity was measured spectrophotometrically (Shimadzu UV-160 spectrophotometer, Shimadzu Scientific Instruments, Kyoto, Japan) by monitoring the rate of H_2_O_2_ decomposition at 240 nm, since H_2_O_2_ absorbs light at this wavelength [40]. The amount of enzyme activity that decomposed 1 mmol of H_2_O_2_ per minute per mg protein at 25 °C and pH 8.0 was defined as one unit of activity.

GPx activity was determined by the GSH-dependent reduction in t-butyl hydroperoxide, using a modified assay described by Paglia and Valentine [41], based on the measurement of NADPH oxidation at 340 nm (Shimadzu UV-160 spectrophotometer, Shimadzu Scientific Instruments, Kyoto, Japan). One unit of GPx activity is defined as the amount of enzyme needed to oxidize 1 nmol NADPH per min per mg protein at 37 °C and pH 7.0.

The activity of GR was measured spectrophotometrically (Shimadzu UV-160 spectrophotometer, Shimadzu Scientific Instruments, Kyoto, Japan) using an assay based on NADPH oxidation and GSSG reduction [42]. One unit of enzyme activity was defined as 1 nmol of NADPH oxidized per min, per mg protein at 25 °C and pH 7.4. Enzyme activities were expressed as U per milligram of protein.

SOD activity was determined using the adrenaline method [43] based on the increase in absorbance at 480 nm (Shimadzu UV-160 spectrophotometer, Shimadzu Scientific Instruments, Kyoto, Japan). One unit of enzyme activity was defined as the amount of the enzyme necessary to reduce the rate of adrenalin auto-oxidation by 50%, at pH 10.2. For the determination of SOD2 activity, the assay was performed after pre-incubation with 8 mM KCN. The SOD1 activity was calculated as the difference between total SOD and SOD2 activities.

The concentrations of total –SH groups were measured according to Ellman’s protocol [44], optimized for a microtiter plate. The principle of the reaction is based on a thiol exchange reaction forming a thiol-5-thio-2-nitrobenzoic acid adduct and a release of one equivalent of 5-thio-2-nitrobenzoic acid, which is quantified by measuring the absorbance of visible light at 412 nm with a Multiskan Spectrum spectrophotometer (Thermo Fisher Scientific Oy, Vantaa, Finland).

To determine the concentration of non-protein –SH groups, proteins were precipitated with sulfosalicylic acid, and the supernatant was used for reaction with Ellman’s reagent. The concentrations of free protein –SH groups were calculated by subtracting the concentrations of non-protein –SH groups from the concentrations of total –SH groups.

### 4.4. Data Analysis and Statistical Procedures

Data recording and analysis were performed using SPEL Advanced Kimograph v2.94 software (Experimetria Ltd. and LogiRex Software Laboratory, Budapest, Hungary). Statistical analyses were performed according to the protocols described by Hinkle [45], using STATISTICA 12.0 software (StatSoft, Inc., Tulsa, OK, USA). The effects of treatments on ileum contractions were calculated as percentages of control. Each data value is expressed as the amplitude of contraction in percentages ± SEM. The effect of the treatment on ileum contractility was tested by two-way ANOVA (factors—type of treatment and concentration of treatment) on logarithmically transformed data and post hoc compared by Tukey’s unequal HSD test (significance—*p* < 0.05). Sigmoid concentration-response curves for carmustine treatment were fitted according to Boltzmann functions (the concentration axis was linear), and the concentration required for EC50 was calculated. Fitted curves were compared using an F-test. EC50 values were compared using the *t*-test (significance: *p* < 0.05). The activities of the antioxidant enzymes were compared by one-way ANOVA followed by Tukey’s HSD post hoc test (significance—*p* < 0.05).

## 5. Conclusions

Our results show that the significant inhibition of glutathione reductase (GR) activity by BCNU led to a contractility reduction in the model of an isolated ex vivo electrically stimulated ileum. Moreover, although the external additions of GSH and GSSG returned the GR activity to control levels, they also caused contractility reductions in a dose-dependent manner. The dose dependent contractility reduction was characteristic of both GSH and GSSG, suggesting that the cellular glutathione turnover and ratio are important, but that direct effects of both GSH and GSSG are also at play, since both GSH and GSSG reduce the contractility even in the untreated ileum, suggesting that the inhibitory effect is not specific to redox cycling or GR inhibition.

## Figures and Tables

**Table 1:** Correlation analyses between the levels of ileum amplitude and antioxidant components at the end of the treatment in both carmustine (BCNU)-treated (below the diagonal) and control R ileum samples (above the diagonal). GR—glutathione reductase, GPx—glutathione peroxidase, CAT—catalase, tSH—total sulfhydryl groups, npSH—non-protein sulfhydryl groups. Amplitude levels were shown as both ratio to control percentage and logarithmic transformed percentage data. Pearson’s correlation coefficients and probability (*p*) are shown (*p* < 0.05 is considered significant and colored red).

	Correlations, BCNU—Below the Diagonal, R—Above the Diagonal						
	Amplitude	Amplitude (ln)	GR	GPx	CAT	tSH	npSH
Amplitude		0.9926 *p* = 0.001	−0.6425 *p* = 0.242	−0.3135 *p* = 0.608	−0.4347 *p* = 0.465	−0.0541 *p* = 0.931	−0.5752 *p* = 0.310
Amplitude (ln)	0.9205 *p* = 0.001		−0.6477 *p* = 0.237	−0.2096 *p* = 0.735	−0.4202 *p* = 0.481	−0.0060 *p* = 0.992	−0.4990 *p* = 0.392
GR	−0.1384 *p* = 0.744	0.0466 *p* = 0.913		0.4585 *p* = 0.437	0.8664 *p* = 0.057	−0.1634 *p* = 0.793	0.7322 *p* = 0.159
GPx	−0.3065 *p* = 0.460	−0.3875 *p* = 0.343	0.0039 *p* = 0.993		0.4949 *p* = 0.397	0.1443 *p* = 0.817	0.8899 *p* = 0.043
CAT	−0.4101 *p* = 0.313	−0.3675 *p* = 0.371	−0.3129 *p* = 0.450	0.7242 *p* = 0.042		0.2818 *p* = 0.646	0.7967 *p* = 0.107
tSH	−0.4457 *p* = 0.268	−0.3246 *p* = 0.433	−0.0661 *p* = 0.876	0.7009 *p* = 0.053	0.6710 *p* = 0.068		0.2882 *p* = 0.638
npSH	−0.7347 *p* = 0.038	−0.7361 *p* = 0.037	0.1170 *p* = 0.783	0.6841 *p* = 0.061	0.5944 *p* = 0.120	0.6377 *p* = 0.089	

**Table 2:** Correlation analyses between the levels of ileum amplitude at the end of both reduced glutathione (GSH) and oxidized glutathione (GSSG) treatment and antioxidant components in ileum samples. GR—glutathione reductase, GPx—glutathione peroxidase, CAT—catalase, SOD1—copper–zinc superoxide dismutase, SOD2—manganese superoxide dismutase. Individual amplitude level was expressed as percentage to corresponding control (considered as 100%). Pearson’s correlation coefficients and probability are shown (*p* < 0.05 is considered as significant).

Variable	GSH Treated—Below the Diagonal, GSSG—Above the Diagonal					
	Amplitude	GR	GPx	CAT	SOD1	SOD2
Amplitude (%)		0.1602	0.4588	0.2451	−0.3456	0.1449
		*p* = 0.732	*p* = 0.300	*p* = 0.596	*p* = 0.448	*p* = 0.757
GR	−0.5116		0.5727	0.2742	0.3487	0.5667
	*p* = 0.159		*p* = 0.179	*p* = 0.552	*p* = 0.443	*p* = 0.185
GPx	−0.3205	0.5001		0.2301	−0.3246	0.7283
	*p* = 0.400	*p* = 0.170		*p* = 0.620	*p* = 0.477	*p* = 0.063
CAT	−0.4390	0.4703	0.1557		−0.5865	−0.0919
	*p* = 0.237	*p* = 0.201	*p* = 0.689		*p* = 0.166	*p* = 0.845
SOD1	0.1118	0.0535	0.6127	−0.4777		0.1519
	*p* = 0.775	*p* = 0.891	*p* = 0.079	*p* = 0.193		*p* = 0.745
SOD2	0.1541	0.2548	0.0930	−0.0288	−0.2984	
	*p* = 0.692	*p* = 0.508	*p* = 0.812	*p* = 0.941	*p* = 0.435	

## Data Availability

The data presented in this study are openly available in the Digital. Repository of Archived Publications of the Institute for Biological Research “Siniša Stanković” at https://radar.ibiss.bg.ac.rs/handle/123456789/8158 (accessed on 29 July 2026).

## Abbreviations

The following abbreviations are used in this manuscript:

GSH	Reduced glutathione
GSSG	Oxidized glutathione
BCNU	Carmustine
EC50	Half maximal effective concentration
GR	Glutathione reductase
CuZnSOD	Copper-zinc superoxide dismutase (SOD1)
CAT	Catalase
GPx	Glutathione peroxidase
NAD^+^	Nicotinamide adenine dinucleotide
NADP^+^	Nicotinamide adenine dinucleotide phosphate
HIFs	Hypoxia-inducible factors
NF-kB	Nuclear factor-kB
H_2_S	Hydrogen sulfide
MnSOD	Manganese superoxide dismutase (SOD2)
–SH	Thiol group or sulfhydryl group
ROS	Reactive oxygen species
H_2_O_2_	Hydrogen peroxide
